# Epilepsy associated with *SYNGAP1* gene variants: clinical features of six cases and a literature review

**DOI:** 10.3389/fped.2026.1789561

**Published:** 2026-04-28

**Authors:** Wenqian Zhang, Yuan Wang, Kaili Xu, Li Wang, Ying Wang, Guohong Chen, Yanli Ma

**Affiliations:** Department of Neurology, Children’s Hospital Affiliated to Zhengzhou University/Henan Children’s Hospital/Zhengzhou Children’s Hospital, Zhengzhou, Henan, China

**Keywords:** developmental delay, epilepsy, genetic diagnosis, *SYNGAP1*, whole exome sequencing

## Abstract

**Objective:**

To summarize the clinical characteristics, treatment response, and prognosis of epilepsy associated with *SYNGAP1* gene variants.

**Methods:**

Clinical and genetic data of six children diagnosed with SYNGAP1-related epilepsy at the Children's Hospital Affiliated to Zhengzhou University between November 2019 and February 2025 were retrospectively analyzed.

**Results:**

Among the six patients (four males and two females), the median age at seizure onset was 2 years and 8 months. All patients showed moderate to severe motor and language developmental delay, with prominent language impairment. Seizure types were heterogeneous, mainly including myoclonic seizures, eyelid myoclonia with or without absence seizures, myoclonic–atonic seizures, and absence seizure. Two patients had a history of febrile seizures, and four had identifiable seizure triggers. Electroencephalography revealed generalized or multifocal epileptiform discharges in all patients. Genetic analysis revealed that all six variants were *de novo*, involving five distinct variant sites, three of which were previously unreported. Variant types included three nonsense mutations, two frameshift mutations, and one missense mutation. Five of the six patients achieved seizure control or marked seizure reduction with valproate treatment, but seizures tended to recur after drug withdrawal. Among them, three patients achieved seizure freedom after combination therapy with levetiracetam, and two patients with drug-resistant epilepsy achieved seizure control after the addition of clobazam.

**Conclusions:**

Myoclonic seizures, absence seizures, and eyelid myoclonia are common in *SYNGAP1*-related epilepsy. Valproate is generally effective, but combination therapy is often required. Neurodevelopmental impairment shows limited improvement despite seizure control.

## 1 Introduction

*SYNGAP1* is located on the short arm of chromosome 6 (6p21.3) and encodes synaptic Ras GTPase-activating protein (SynGAP), which was first reported in 1998 (1). This gene plays a critical role in neurodevelopment and glutamatergic neurotransmission and is essential for synaptic plasticity and neuronal signal transduction. Loss-of-function variants in *SYNGAP1* lead to abnormal neuronal excitability, thereby predisposing individuals to epilepsy (2, 3). Epilepsy associated with *SYNGAP1* variants is most commonly classified as idiopathic generalized epilepsy and is frequently accompanied by intellectual disability, global developmental delay, epileptic seizures, autism-like features, and other behavioral abnormalities. In this study, we retrospectively analyzed the clinical characteristics of children with *SYNGAP1* variant–associated epilepsy treated in the Department of Neurology at Henan Children's Hospital between 2019 and 2025. By integrating clinical manifestations, laboratory findings, and molecular genetic features with a review of the literature, we aimed to explore genotype–phenotype correlations and to improve clinicians' awareness of this condition, facilitating early recognition and appropriate management.

## 2 Materials and methods

### 2.1 Study population

A retrospective analysis was conducted on the clinical data of six children with SYNGAP1-related epilepsy who were diagnosed and treated in the Pediatric Neurology Department at Henan Children's Hospital between November 2019 and February 2025. All cases were identified through next-generation sequencing and confirmed by Sanger sequencing. This study was approved by the Ethics Committee of Henan Children's Hospital (2023-H-K40), and informed consent was obtained from the patients' legal guardians.

### 2.2 Clinical data collection

Clinical data were obtained by reviewing inpatient and outpatient medical records, combined with follow-up visits and telephone interviews. Information collected included sex, age at onset, follow-up duration, developmental status, age at first seizure, antiepileptic drug usage, seizure triggers, and daily behavior. Treatment outcomes, including seizure control and growth and developmental status, were also recorded. Auxiliary examination results, including electroencephalography (EEG), brain magnetic resonance imaging (MRI), blood and urine metabolic screening, and genetic testing results, were summarized.

### 2.3 Whole-exome and sanger sequencing

Peripheral venous blood samples (2 mL each) were collected from the patients and placed in EDTA anticoagulant tubes. Genomic DNA was extracted using the QIAamp® Blood Mini Kit (Qiagen, Hilden, Germany) following the manufacturer's instructions. The DNA was then fragmented using a Covaris ultrasonic device, followed by library preparation. High-throughput sequencing was performed on the Illumina HiSeq 2000 platform to generate 150-base pair paired-end reads. After quality control, the sequencing data were aligned to the human reference genome GRCh37/hg19, and candidate variants were filtered. Specific primers were designed for the candidate variants for PCR amplification, and Sanger sequencing was performed using the 3730xl sequencer (Thermo Fisher Scientific, USA) to validate the variants.

### 2.4 Bioinformatic analysis

Candidate variants were queried in the Human Gene Mutation Database (HGMD, https://www.hgmd.cf.ac.uk/ac/index.php), the Online Mendelian Inheritance in Man database (OMIM, https://omim.org/), and the ClinVar database (https://www.ncbi.nlm.nih.gov/clinvar/). Their allele frequencies in the general population were checked using the Exome Aggregation Consortium database (ExAC, http://exac.broadinstitute.org/), the 1000 Genomes Project database (http://www.internationalgenome.org/), and the Genome Aggregation Database (gnomAD, https://gnomad.ncbi.nlm.nih.gov/). Candidate variants were classified and their pathogenicity evaluated according to the 2015 guidelines of the American College of Medical Genetics and Genomics (ACMG) (4).

### 2.5 Literature review

Using “SYNGAP1” as the search term, relevant literature in the NCBI PubMed database was retrieved from its inception to February 2025. Reported cases of Chinese children (a total of 67 patients with *SYNGAP1*-related epilepsy) were retrospectively analyzed to systematically summarize their clinical features and genetic findings.

## 3 Result

### 3.1 Clinical features of the cases

**Figure 1:**
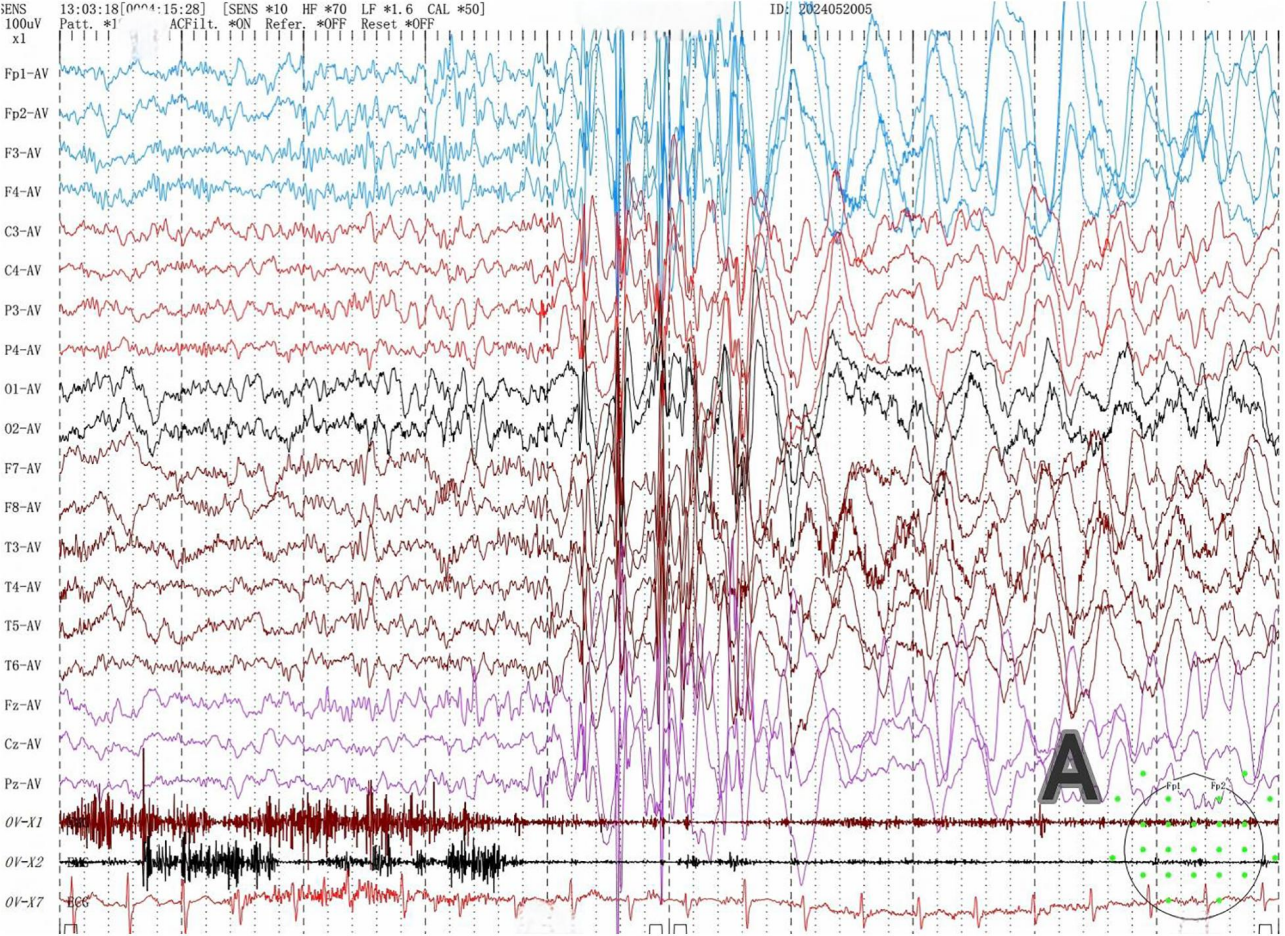
Myoclonic seizures.

**Figure 2:**
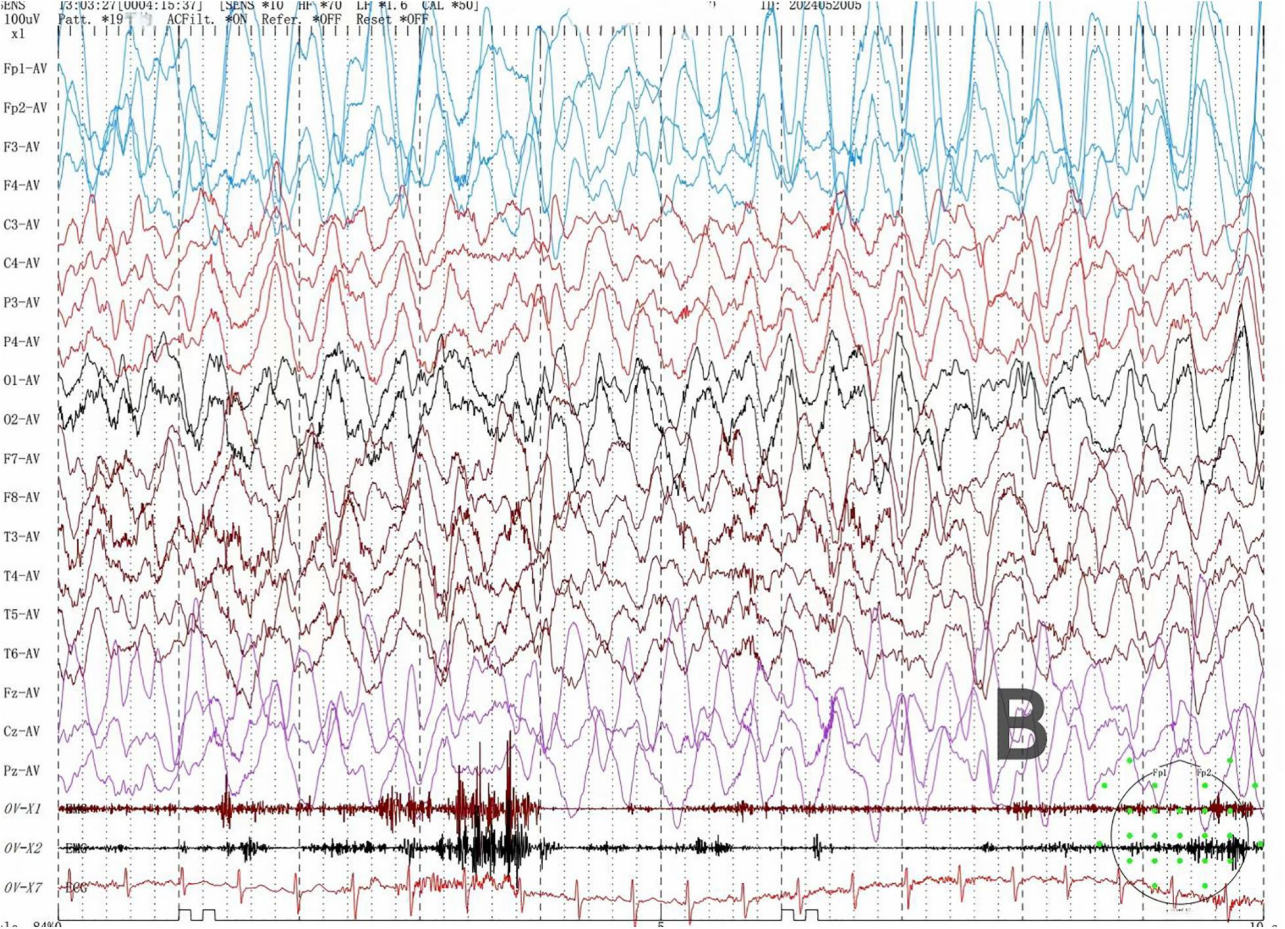
Atypical absence seizures.

**Figure 3:**
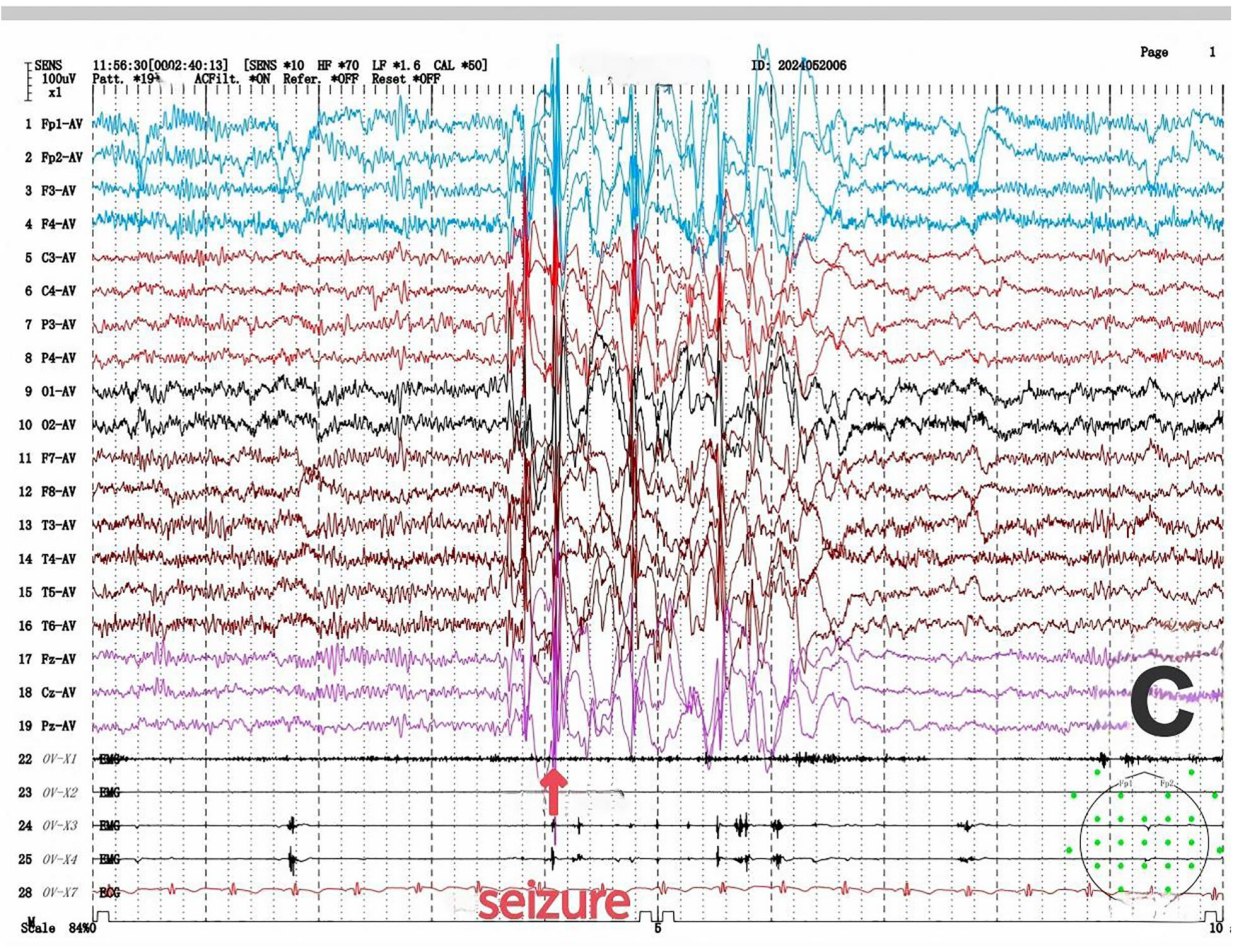
Eyelid myoclonia.

Among the six children with SYNGAP1-related epilepsy, four were male and two were female. The age at first seizure ranged from 1 year 7 months to 5 years 3 months, with a mean age of 2 years 9 months and a median age of 2 years 8 months. Four patients had identifiable seizure triggers: two were related to eating, drinking, or emotional excitement, one to drowsiness, and one was fever-induced. One patient exhibited developmental regression following seizure recurrence. All patients had unremarkable birth histories. All patients underwent video EEG, which showed abnormal findings in all cases. Background activity was slowed, and interictal recordings revealed multifocal (bilateral occipital and temporal regions) and generalized spikes or spike-slow wave discharges. Multiple seizure types were observed in all six patients, including myoclonic seizures (3 cases, Figure 1), myoclonic-atonic seizures (1 case), and absence seizures (3 cases, including 2 atypical (Figure 2) and 1 typical absence seizure), eyelid myoclonia (Figure 3) with or without absence seizures (3 cases). Two patients had a history of febrile seizures. Brain MRI scans were normal in all patients. Metabolic screening of blood and urine revealed reduced free carnitine in two patients, while other parameters, including homocysteine, blood ammonia, and lactate, were within normal limits (Table 1).

**Table 1:** Clinical data of six cases with SYNGAP1 variant–associated epilepsy.

Characteristics	Case 1	Case 2	Case 3	Case 4	Case 5	Case 6
Gender	Male	Male	Female	Male	Male	Female
Age at Onset	2 years 3 months	2 years 3 months	1 years 9 months	5 years 3 months	1 years 7 months	3 years
Seizure Frequency	5-10 times/day	About 10 times/day	About 10 times/day	Several times/day	Several times/day	Dozens of times/day
Seizure Triggers	Fever	Eating, emotional excitement, drowsiness	Emotional excitement	Eating, drinking, tooth brushing	None	None
Seizure Type	AE, EA	MS, AA, EA	MS	MAS	AA, MS	EA
EEG	Slowed background activity with multifocal and generalized spikes and spike-slow wave discharges	Slowed background activity, multifocal and generalized spikes and spike-slow wave discharges predominantly in bilateral occipital and temporal regions	Slowed background activity with multifocal and generalized spikes and spike-slow wave discharges	Slowed background activity, interictal generalized spikes, spike-slow waves, polyspike-slow waves, and spike rhythm	Slowed background activity with paroxysmal 2–3 Hz slow waves in bilateral occipital regions and generalized 2–4 Hz spike-slow wave discharges during sleep	Slowed background activity with spikes and spike-slow wave discharges in bilateral occipital and generalized regions
Growth and Development	Independent walking at 2 years, severe developmental delay	Independent walking at 2 years 3 months, moderate developmental delay	Independent walking at 1 year 5 months, severe developmental delay	Independent walking at 1 year 5 months, mild developmental delay	Independent walking at 1 year 8 months, moderate developmental delay	Independent walking at 1 year 10 months, moderate developmental delay with regression
Gesell Developmental Schedules (DQ)	35	50	27	62	47	-
Autism Spectrum Disorder	Yes, CABS = 20	No, CABS = 12	Yes, CABS = 18	No, CABS = 10, ABC = 30	Yes, ABC = 54SRS−2 = 135	Yes, CBAS = 21
Gait	Unsteady walking	Ataxia	Ataxia	Unsteady walking	Tiptoe walking	Unsteady walking
Maximum Language Ability	2–3 words, cannot form a sentence	Single words, cannot form a sentence	2–4 words, cannot form a sentence	Able to form sentences	1–2 words, cannot form a sentence	2–3 words, cannot form a sentence
Daily Behavior	Irritability and easy anger	Irritability and easy anger	Normal	Irritability and aggressive behavior	Irritability and aggressive behavior	Autism/Autistic features
Blood and Urine metabolic	Normal	Reduced free carnitine	Reduced free carnitine	Normal	Normal	Normal

EM, eyelid myoclonia; EA, eyelid myoclonia with absence; MS, myoclonic seizures; AA, atypical absence seizures; AE, absence seizures; AS, atonic seizures; MAS, myoclonic–atonic seizures; GTCS, generalized tonic–clonic seizures. DQ (developmental quotient) <70, indicates developmental delay; CABS (clancy autism behavior scale) ≥14, strongly indicates autism; ABC (autism behavior checklist) ≥54, strongly indicates autism; SRS-2 (social responsiveness scale second edition) ≥76, strongly indicates autism.

Among the six patients, developmental delay was present both before and after disease onset. Five patients underwent evaluation using the Gesell Developmental Schedules after disease onset, and all five showed DQ (Developmental Quotient) values <70, indicating developmental delay. Based on comprehensive assessment, two patients were classified as having severe developmental delay, two as moderate developmental delay, and one as mild developmental delay. The remaining patient was considered to have moderate developmental delay based on parental report. The age at independent walking ranged from 1 year 5 months to 2 years 3 months, with a median of 1 year 9 months. Language development was markedly delayed, with most patients able to produce only 1–3 words, and only one patient able to speak short sentences. Based on the combined evaluations using the Clancy Autism Behavior Scale (CABS), Autism Behavior Checklist (ABC), and Social Responsiveness Scale Second Edition (SRS-2), four patients were highly suggestive of autism. All six patients had abnormal gait, manifested as unsteady walking or ataxia. Four patients displayed irritability, easy anger, or aggressive behavior. One patient had sleep disturbances, characterized by difficulty falling asleep and frequent awakenings (Table 1).

### 3.2 Genetic diagnosis

To further investigate the potential genetic causes, whole-exome sequencing was performed on the six patients. The results showed that all six children with epilepsy carried SYNGAP1 gene variants, involving a total of five variant sites (case 5 and 6, who are siblings, shared the same variant), all of which were *de novo* mutations (Table 2). The variants included two nonsense mutations, two frameshift mutations, and one missense mutation. According to the ACMG guidelines, all five variants were classified as pathogenic.

**Table 2:** Genetic diagnosis of six cases with SYNGAP1 variant–associated epilepsy.

ID	Location	Nucleotide variant	Amino acid variant	Variant type	Pathogenicity	Previously reported
Case 1	Exon 15	c.3092–3096 del TGCTG	p.M1031Ifs*120	Frameshift mutation	Pathogenic	No
Case 2	Exon 15	c.599T > A	p.L200*,1144	Nonsense mutation	Pathogenic	No
Case 3	Exon 11	c.1729G > C	p.A577P(Ala577Pro)	Missense mutation	Pathogenic	No
Case 4	Exon 4	c.333del	p.Lys114SerfsTer20	Frameshift mutation	Pathogenic	Yes
Case 5	Exon 5	c.427C > T	p.R143X	Nonsense mutation	Pathogenic	Yes
Case 6	Exon 5	c.427C > T	p.R143X	Nonsense mutation	Pathogenic	Yes

Among these five variants, two had been previously reported. The variants carried by patients 1, 2, and 3 (c.3092_3096delTGCTG, c.599T>A, and c.1729G>C) were not found in the HGMD, OMIM, or ClinVar databases. Furthermore, these three variants were absent in the Asian population according to the ExAC, 1,000 Genomes, and gnomAD databases, indicating that they represent novel *de novo* mutations.

### 3.3 Treatment and follow-up

All six patients responded to initial treatment with valproic acid. Complete seizure control was achieved in Cases 1, 3, and 6, while seizure frequency was reduced by more than 75% in Cases 2 and 4, and partially reduced in case 5 (Table 3).

**Table 3:** Treatment and follow-up of six cases with *SYNGAP1* variant–associated epilepsy.

Cases	Treatment	Seizure control	Age at last follow-up	Educational level
Case 1	VPA, LEV, LTG, LBZ	Seizure-free for 4 years 5 months	10 years 1 month	Kindergarten
Case 2	TPM, LEV, VPA, CZP, LBZ	Seizure-free for 1 year	6 years	Not attending school
Case 3	VPA, TPM, LEV	Seizure-free for 5 years 2 months	7 years 2 months	Kindergarten
Case 4	VPA, LEV	Seizure-free for 11 months	6 years 8 months	Kindergarten
Case 5	VPA	Seizure frequency reduced	7 years 9 months	Kindergarten
Case 6	VPA, LEV	Seizure-free for 7 years	14 years 2 months	Dropped out

VPA, valproate; TPM, topiramate; LEV, levetiracetam; LTG, lamotrigine; CZP, clonazepam; LBZ, clobazam.

Among the three patients with complete seizure control, Case 1 experienced seizure recurrence after being seizure-free for 4 years and 5 months. Subsequent combination therapy with levetiracetam and lamotrigine failed to achieve full control, whereas the addition of clobazam resulted in seizure freedom. Case 6 relapsed after discontinuation of antiepileptic medication for 1–2 months following 3 years of seizure freedom; seizure control was regained after the addition of levetiracetam, although language regression was observed. Among the three patients with partial seizure reduction, Case 2 was sequentially treated with topiramate, levetiracetam, valproic acid, lamotrigine, and clonazepam, but seizures remained uncontrolled. Seizure control was achieved after adjustment to combination therapy with valproic acid, levetiracetam, and clobazam. Case 4 showed no obvious clinical seizures after the addition of levetiracetam, while Case 5 did not receive further combination therapy.

Overall, seizures in this cohort were well controlled with combination antiepileptic drug therapy, with the longest seizure-free duration reaching 7 years. However, despite effective seizure control, neurodevelopmental delay did not show significant improvement.

### 3.4 Literature review

To date, a total of 67 cases of *SYNGAP1*-related epilepsy in Chinese patients have been reported, including those in the present study (5, 6). All patients exhibited intellectual disability and developmental delay of varying degrees. The mean age at seizure onset was 29.2 months, ranging from 4 to 82 months. Autism spectrum–like features were reported in 23 patients (34.3%). EEG data were available for 65 patients, of whom 47 (70.1%) showed background slowing. Interictal discharges were predominantly generalized spike–slow wave or polyspike-slow wave patterns, and 5 patients were reported to have definite photosensitivity. Brain MRI revealed no abnormalities in 59 patients (88%), while 8 patients (12%) showed nonspecific changes. Eight patients (12%) demonstrated a clearly increased pain threshold. Seizures were frequently associated with triggering factors. Among 46 patients with documented triggers, seizures were induced by eating in 11 patients (24%), and by emotional excitement or crying in 9 patients (19%). Among the 59 patients with available antiseizure medication records, valproic acid was effective in the majority (51 patients, 86%), and 28 patients achieved complete seizure control with valproic acid monotherapy or combination therapy.

Genetic analysis showed that all 67 patients carried *de novo* variants. The variant types included 27 nonsense variants (40%), 21 frameshift variants (31%), 15 missense variants (22%), 5 splice-site variants (7%), and one 6p21.3 deletion. No clear genotype–phenotype correlation was observed among different SYNGAP1 variant types, and considerable phenotypic heterogeneity was noted even among patients carrying the same variant. No mutational hotspots have been identified; however, variants located in exons 1–4 have been reported to be associated with relatively milder phenotypes (7).

During the VEEG monitoring of Patient 5, an atypical absence seizure followed immediately after a myoclonic seizure. The child presented with slight nodding → mild decrease in responsiveness. Simultaneous electroencephalogram showed generalized high-amplitude spike-and-wave paroxysms (Figure 1) → generalized high-amplitude slow-wave discharges (Figure 2).

In Case 6, video EEG monitoring captured eyelid myoclonic seizures, manifested as rhythmic blinking in the child. Concurrently, generalized moderate to high amplitude spike-and-slow waves and polyspike-and-slow wave discharges were observed, along with bursts on electromyography (EMG) (Figure 3).

## 4 Discussion

As of January 2025, a total of 340 *SYNGAP1* variants have been recorded in the Human Gene Mutation Database (HGMD), among which 285 cases are associated with epilepsy or epileptic encephalopathy. To date, reported *SYNGAP1* variants are distributed across 19 exons and include missense, nonsense, frameshift, and splice-site variants, as well as microdeletions involving the 6p21.3 region. A small number of cases result from balanced chromosomal translocations leading to disruption of the *SYNGAP1* gene (8–11). No mutational hotspots have been identified in either domestic or international reports. In the present study, all six patients with epilepsy carried *de novo SYNGAP1* variants, involving five distinct variant sites. These included two nonsense variants, two frameshift variants, and one missense variant, all classified as pathogenic. Three of these variants have not been previously reported. Cases 5 and 6 were siblings, while both parents were wild type and phenotypically normal, suggesting the possibility of parental germline mosaicism; however, further validation was declined by the family, and the variant origin remains unclear. To date, six cases of *SYNGAP1*-related germline mosaicism have been reported, including three maternal, two paternal, and one of unknown origin (12).

Previous studies suggest a certain genotype-phenotype correlation in *SYNGAP1*-related disorders. Variants affecting exons 1–4 are generally associated with milder clinical phenotypes, whereas variants involving exons 8–15 are more frequently associated with severe phenotypes (8). This may be related to the presence of multiple SynGAP1 isoforms, one of which lacks exons 1–4, as well as differences in nonsense-mediated mRNA decay. In the present cohort, one patient carried a variant in exon 4 and exhibited relatively preserved cognitive function, the ability to produce short sentences, and better seizure control, consistent with previous reports.

*SYNGAP1* encodes SynGAP1, a key regulator of intracellular biochemical signaling in neurons that plays a critical role in neuronal function. SynGAP1 is an abundant component of the postsynaptic density (PSD) of excitatory glutamatergic neurons and is also an essential component of the N-methyl-D-aspartate receptor (NMDAR) complex. By regulating AMPA receptors (AMPARs), SYNGAP1 functions as a critical Ras-GAP protein enriched at excitatory synapses and is essential for synaptic development, structure, function, and plasticity (13). Recent studies have shown that, in addition to acting as a negative regulator of G-protein signaling through its GAP enzymatic activity, SynGAP1 also exerts important structural roles through interactions with postsynaptic density proteins (14). Induced neurons lacking SynGAP expression exhibit accelerated dendritic morphogenesis, increased accumulation of postsynaptic markers, premature onset of synaptic activity, enhanced excitatory synaptic strength, and early emergence of network activity. These abnormalities are frequently associated with human intellectual disability and epilepsy (15). SYNGAP1 mRNA and protein are predominantly expressed in the brain, particularly in the forebrain regions such as the cortex, hippocampus, and olfactory bulb. Most pathogenic *SYNGAP1* variants result in loss of function, leading to haploinsufficiency, which manifests clinically as epilepsy, intellectual disability, psychomotor developmental delay, autism spectrum features, and other behavioral abnormalities (2). All patients in this study exhibited moderate to severe motor and language developmental delay, with language impairment being particularly prominent, consistent with the typical phenotype of *SYNGAP1*-related encephalopathy described by Vlaskamp et al. (16).

Previous reports indicate that the age of seizure onset in *SYNGAP1*-related epilepsy ranges from 6 months to 7 years, with a peak between 2 and 3 years of age (3, 7, 16). Seizure types are predominantly generalized and highly heterogeneous, including myoclonic seizures with or without falls, eyelid myoclonia, generalized tonic–clonic seizures, myoclonic absence seizures, absence seizures, atonic seizures, and myoclonic–atonic seizures. Focal seizures and epileptic spasms are less common (3, 16). Kimberly et al. summarized 123 cases from the United States and found that absence seizures and atonic seizures were the most common manifestations, with absence seizures observed in 54% of patients (34% typical and 20% atypical) and atonic seizures in 34% (12). Epileptic phenotypes often evolve over time; up to 35% of patients exhibit clear evolution, such as eyelid myoclonia progressing to myoclonic epilepsy and subsequently to atonic seizures (2). Seizures are typically frequent but brief, often lasting only a few seconds. In the present study, seizure onset ranged from 1 year 7 months to 5 years 3 months, with a median age of 2 years 8 months. Seizure types were mainly eyelid myoclonia with or without absence, myoclonic seizures, and absence seizures, with one patient exhibiting myoclonic-atonic seizures, largely consistent with previous reports. Although atonic seizures are commonly reported in the literature, two patients in this cohort experienced sudden loss of tone and falls without corresponding ictal EEG changes, precluding definitive classification as atonic seizures.

SYNGAP1-related epilepsy is characterized by prominent reflex seizure features (16–18). Studies indicate that 25%–54% of patients experience seizures triggered by eating, chewing, emotional excitement, or crying, primarily related to oral motor activity or orofacial sensory stimulation. The most common reflex seizure types include eyelid myoclonia with absence and seizures with myoclonic or atonic components. Photosensitivity has also been reported. Approximately half of patients exhibit eye-closure sensitivity or fixation-off sensitivity, with seizures or epileptiform discharges triggered by blinking or eye closure (16–18). In the present cohort, seizures were triggered by eating in two patients and by emotional excitement in two patients; only one patient exhibited eye-closure-induced seizures, which is lower than previously reported and may be attributed to the small sample size, recall bias, and limited patient cooperation.

Previous studies of 133 patients with SYNGAP1 variants reported generalized spike-and-wave discharges in 63% of EEGs and generalized slowing in 38%, with only 8% showing focal abnormalities (12). Among 65 Chinese cases with available EEG data, 70.1% exhibited background slowing with generalized spike-and-wave or polyspike-and-wave discharges. In the present study, all six patients underwent video EEG monitoring and demonstrated background slowing with multifocal (bilateral occipital and temporal) and generalized spike or spike-and-wave discharges, consistent with previous findings. These EEG features suggest that SYNGAP1 variants should be considered when background slowing with generalized spike-and-wave activity is observed.

Developmental delay or intellectual disability is nearly universal in SYNGAP1-related epilepsy, with most patients exhibiting moderate to severe impairment (2, 3). Approximately 68% of patients have behavioral problems such as aggression, self-injury, or irritability; 68% exhibit autism spectrum features; 61% have sleep disturbances; and 47% show ataxia or abnormal gait (12). In this study, all six patients demonstrated developmental delay prior to seizure onset. Three patients underwent Gesell developmental assessment and were classified as having moderate to severe impairment. Language delay was particularly severe, with most patients limited to one to three words and only one patient able to produce short sentences. Five patients exhibited autism spectrum features, all six had abnormal gait, and four had irritability or aggressive behavior, findings largely consistent with previous reports. Only one patient exhibited sleep disturbances, which may reflect the small sample size and lack of systematic sleep assessment. Notably, two patients demonstrated decreased free carnitine levels on metabolic screening, a finding not previously reported and warranting further investigation.

Regarding treatment, approximately half of patients can achieve seizure control with monotherapy, although recurrence may occur after seizure-free periods ranging from 6 months to 7 years; the remaining patients require combination therapy with two or more antiseizure medications (ASMs) (19). Kimberly et al. reported that clobazam, valproic acid, lamotrigine, and levetiracetam are commonly used or reserved ASMs (12). In the present cohort, five patients responded well to valproic acid, highlighting its importance as a broad-spectrum ASM. However, relapse following drug withdrawal suggests that long-term treatment may be necessary in *SYNGAP1*-related epilepsy. Three patients achieved seizure freedom after the addition of levetiracetam, and two patients with refractory epilepsy achieved final seizure control after clobazam was added, supporting its potential efficacy. Topiramate showed limited efficacy and notable adverse effects in this cohort and is therefore not recommended. Other treatments, including ketogenic diet therapy and surgical interventions such as vagus nerve stimulation or corpus callosotomy, have shown limited benefit. Cannabidiol has been reported to reduce seizure frequency by 80%–90%, but its limited availability has restricted its use domestically (20). Preclinical studies suggest that the AMPAR antagonist perampanel may be effective by reducing glutamate-mediated neuronal hyperexcitability (2, 21). Rosuvastatin has also shown partial efficacy, possibly through modulation of Ras signaling pathways (22, 23). In 2023, Yang et al. identified a variable splicing site in intron 10 of SYNGAP1; deletion of this site increased SynGAP1 protein expression and significantly improved neurological function in mice, suggesting a promising therapeutic target for future precision treatments (24).

## 5 Conclusion

Absence seizures, myoclonic seizures, and eyelid myoclonia with or without absence are common phenotypes of epilepsy associated with *SYNGAP1* gene variants. Valproic acid is effective for seizure control in most patients; however, monotherapy is often insufficient, and combination therapy is frequently required. Adjunctive treatment with levetiracetam or clobazam appears to be beneficial, with clobazam providing additional efficacy in patients with drug-resistant epilepsy. Neurodevelopmental impairment typically shows little improvement after seizure remission, suggesting that the impact of *SYNGAP1* variants on neurodevelopment may be largely independent of seizure activity. In children with early-onset epilepsy accompanied by developmental delay, particularly those with EEG background slowing and generalized or multifocal epileptiform discharges, *SYNGAP1* variants should be considered-especially when seizures exhibit reflex features. Early genetic testing is therefore recommended to establish a definitive diagnosis and to guide treatment strategies and genetic counseling.

## Data Availability

The raw sequence data reported in this paper have been deposited in the Genome Sequence Archive (Genomics, Proteomics & Bioinformatics 2025) in National Genomics Data Center (Nucleic Acids Res 2025), China National Center for Bioinformation / Beijing Institute of Genomics, Chinese Academy of Sciences (GSA-Human: subHRA028210, and BioProject number:PRJCA062232) that are publicly accessible at https://ngdc.cncb.ac.cn/gsa-human.
